# A spiking neuron network model for the delayed motion direction discrimination task

**DOI:** 10.1186/1471-2202-16-S1-P281

**Published:** 2015-12-18

**Authors:** Liu-Tao Yu, Si Wu, Da-Hui Wang

**Affiliations:** 1 School of Systems Science, Beijing Normal University, Beijing 100875, China; 2 State Key Laboratory of Cognitive Neuroscience & Learning, Beijing Normal University, Beijing 100875, China

## 

**Figure 1:**
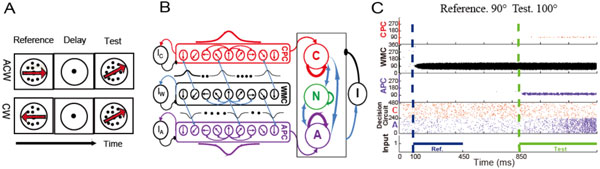
**A. DMD task: either *ACW *or *CW *has to be reported in relation to the reference**. **B**. Network structure. Pyramid cells of WMC (CPC and APC) are arranged on a circle according to their preferred motion directions and with rotation invariant Gaussian shaped lateral connections. Note that from WMC to CPC and APC connectivity is offset by -45°/+45°. Right box indicates the decision making network. **C**. Network activity during the DMD task. Reference (90°) is applied to the WMC (blue line), while the test (100°) is applied to CPC and APC (green line). Due to the connectivity structure (see B), the group A of the decision circuit activates stronger than group C indicating a correct *ACW *decision.

Drawing the correct decision in a given situation usually depends on past experiences. While the neural basis of decision making *per se *has been studied extensively, how a memory of a previous situation could influence a later decision is less clear. One behavioral paradigm for investigating decision making in relation to an earlier reference is the delayed motion direction discrimination task (DMD) [1]. In this task, a variable reference direction is first presented to the subject by displaying a coherent random dot kinematograms (RDK). After memorizing, a delayed presentation of the test direction (RDK) follows and the subject needs to report whether the test direction is *clockwise *(*CW*) or *anticlockwise *(*ACW*) rotated in relation to the reference direction (Figure 1A). Although phenomenological models have been proposed, the underlying neural mechanism remains relatively unknown. Here, we hypothesize that a mechanism based on the idea of asymmetric connections similar to those used in neural networks explaining angular path integration in the head-direction system [2], might explain the behavioral results.

To test this hypothesis, we build a spiking neuron network model and investigate whether it can solve the DMD task. The network consists of three parts (Figure 1.B): 1) a working memory circuit (WMC) 2) two information extraction circuits, referred to as clockwise-preferred circuit (CPC) and anticlockwise-preferred circuit (APC) respectively, and 3) a decision-making circuit. At the core of the network is the assumption of an asymmetric offset and rotational invariance of the connectivity profile. The former ensures that CPC (or APC) has stronger response when the test direction is *CW *(or *ACW*) rotated with respect to the reference direction and the latter guarantees the feasibility of the network under variable references. The simulations demonstrate that the proposed network is indeed capable of solving the DMD task (Figure 1.C) showing a similar detection threshold (2°) as previous behavioral data [3]. Furthermore, the model correctly predicts that with higher similarity of reference and test direction or lower coherence level of the RDK, the performance gets worse (lower accuracy and longer reaction time). Our results suggest a possible neural mechanism for memory-guided decision making.
